# Day-7 caloric intake as an early dynamic prognostic marker for subsequent mortality in older patients with dysphagia: a landmark cohort analysis

**DOI:** 10.3389/fnut.2026.1887101

**Published:** 2026-08-26

**Authors:** Jianxian Zhang, Xiaojing Yun, Yan Xue

**Affiliations:** 1 Department of Gastrointestinal Surgery, The Second People’s Hospital of Liaocheng, Liaocheng, Shandong, China; 2 Department of Gastroenterology, The Second People’s Hospital of Liaocheng, Liaocheng, Shandong, China

**Keywords:** caloric intake, dysphagia, elderly population, landmark analysis, mortality

## Abstract

**Background:**

Early nutritional status may influence prognosis in older adults with dysphagia receiving artificial nutrition. However, the predictive value of caloric intake shortly after initiation of enteral or parenteral nutrition remains unclear. This study aimed to investigate the association between day-7 caloric intake and subsequent all-cause mortality in older Japanese patients with dysphagia and assess its potential as an early dynamic prognostic marker.

**Methods:**

We conducted a day-7 landmark cohort analysis of 246 older adults who survived beyond 7 days after initiation of percutaneous endoscopic gastrostomy (PEG) or total parenteral nutrition (TPN). Day-7 caloric intake was analyzed both as a continuous variable (per 100 kcal/day) and categorically (<900 vs. ≥900 kcal/day). Multivariable Cox proportional hazards models adjusted for age, sex, nutritional route, severe dementia, aspiration pneumonia, chronic heart failure, serum albumin, hemoglobin, C-reactive protein, and Clinical Frailty Scale were used to estimate hazard ratios (HRs) for subsequent all-cause mortality. Kaplan–Meier survival curves, subgroup analyses, and time-dependent receiver operating characteristic (ROC) curves at 365 days were applied to evaluate robustness and predictive discrimination.

**Results:**

Higher day-7 caloric intake was consistently associated with lower subsequent all-cause mortality across all models. Each 100 kcal/day increase was associated with lower subsequent mortality in the fully adjusted model (HR, 0.82; 95% CI 0.72–0.93; *P* = 0.002). In secondary categorical analysis, patients receiving ≥900 kcal/day had lower mortality than those with <900 kcal/day (HR, 0.47; 95% CI 0.26–0.83; *P* = 0.010). Kaplan–Meier curves showed higher survival probability in the high-intake group (*P* = 0.01), and subgroup analyses showed no statistically significant interactions across prespecified strata (all *P* for interaction > 0.05). Time-dependent ROC analysis showed a modest increase in AUC after adding day-7 caloric intake to the baseline model, indicating improved predictive discrimination.

**Conclusion:**

Among older patients with dysphagia who survived beyond the day-7 landmark, higher recorded day-7 caloric intake was associated with lower subsequent all-cause mortality. Day-7 caloric intake may therefore serve as an early dynamic prognostic marker for risk stratification in this population. Future multicenter prospective studies are warranted to validate these findings and explore whether early nutritional optimization can improve survival and patient-centered outcomes.

## 1 Introduction

Oropharyngeal dysphagia is a common and clinically important geriatric syndrome (1). Normal swallowing requires coordinated neuromuscular control, preserved cognition, effective airway protection, and adequate functional reserve; impairment in these domains may result in inefficient bolus transit, aspiration, and insufficient nutritional intake (2, 3). Older adults are particularly vulnerable because aging is frequently accompanied by sarcopenia, frailty, multimorbidity, cognitive impairment, and neurological diseases such as stroke, dementia, Parkinson’s disease, and neuromuscular disorders (4, 5). Recent systematic reviews and meta-analyses have shown that oropharyngeal dysphagia is prevalent across hospital, rehabilitation, nursing home, and palliative care settings, and is associated with serious adverse outcomes, including malnutrition, dehydration, pneumonia, institutionalization, prolonged hospitalization, readmission, and mortality (2, 3, 6). These adverse outcomes are especially relevant among very old patients with severe dysphagia who require artificial nutritional support, in whom clinical decisions must balance survival, infection risk, nutritional achievement, recovery potential, and goals of care (7, 8). Therefore, early identification of clinically accessible prognostic markers is essential for risk stratification and individualized management in this vulnerable population.

Artificial nutritional support is commonly used when oral intake is unsafe or insufficient in older patients with severe dysphagia. Percutaneous endoscopic gastrostomy (PEG) is usually considered when the gastrointestinal tract is functional, whereas total parenteral nutrition (TPN) may be selected when enteral feeding is not feasible or poorly tolerated. In the original Japanese cohort from which the present Dryad dataset was derived, PEG was associated with longer survival, a higher incidence of severe pneumonia, and a lower incidence of sepsis compared with TPN (7). Subsequent secondary analyses of this cohort have mainly focused on baseline nutritional and inflammatory biomarkers, including serum albumin, the C-reactive protein-to-albumin ratio, the prognostic nutritional index, the controlling nutritional status score, and composite inflammation–nutrition indices (9–15). These studies suggest that prognosis in older patients with dysphagia is influenced by the interplay of malnutrition, systemic inflammation, immune dysfunction, frailty, and comorbidity burden. However, most available prognostic markers are static baseline indicators and do not capture early nutritional achievement or treatment tolerance after artificial nutritional support. Because energy intake is a core component of geriatric nutritional care and should be individualized according to nutritional status, disease severity, physical activity, and tolerance (16), caloric intake shortly after PEG or TPN initiation may reflect early clinical stability and recovery potential. Therefore, day-7 caloric intake may serve as an early dynamic prognostic marker rather than merely a measure of nutritional exposure.

A key methodological issue is that day-7 caloric intake was measured after the initiation of artificial nutritional support and therefore could not be treated as a conventional baseline covariate. Patients must survive long enough to have this exposure assessed; thus, including deaths before day 7 would introduce temporal inconsistency and potential immortal time bias. Landmark analysis provides an appropriate framework for this setting by defining a fixed landmark time, excluding patients who experienced the event before that time, and evaluating subsequent outcomes only among patients who remained at risk at the landmark time (17, 18). This approach allows day-7 caloric intake to be interpreted as information available at a post-procedural reassessment point.

Accordingly, this secondary cohort analysis aimed to evaluate the association between day-7 caloric intake and subsequent all-cause mortality in older Japanese patients with dysphagia who survived beyond 7 days after artificial nutritional support. We further assessed whether adding day-7 caloric intake to a baseline clinical model improved mortality risk prediction. By focusing on a dynamic post-procedural marker and using a landmark design, this study may help clarify whether early caloric intake provides clinically meaningful prognostic information beyond conventional baseline characteristics.

## 2 Materials and methods

### 2.1 Study design and data source

This study was a secondary analysis of a publicly available cohort dataset from the Dryad repository (DOI: 10.5061/dryad.gg407h1), which included older adults with dysphagia receiving percutaneous endoscopic gastrostomy (PEG) or total parenteral nutrition (TPN). Ethical approval for the original study was obtained from the Institutional Review Board of Miyanomori Memorial Hospital, and informed consent was waived due to the use of anonymized retrospective data. This secondary analysis was conducted in accordance with relevant ethical standards, including the Declaration of Helsinki. According to the original cohort report, clinical information was retrospectively extracted from medical records. The public dataset did not specify whether the day-7 caloric intake variable was derived from EHR orders, nursing flowsheets, pump logs, dietitian notes, or other source documents. Because the present study used the released analytic dataset, no independent chart re-extraction or interrater validation was performed.

### 2.2 Landmark cohort definition

The landmark time was defined as day 7 after initiation of PEG feeding or TPN. Of 253 older adults receiving PEG or TPN, seven patients with survival time ≤7 days were excluded, leaving 246 patients in the day-7 landmark cohort. Follow-up time was recalculated from the landmark point as the original survival time minus 7 days. Day-7 caloric intake and all covariates included in the final multivariable models had no missing values. Because this was a secondary analysis of a fixed publicly available dataset, no formal *a priori* sample size calculation was performed.

### 2.3 Exposure and outcome

The primary exposure was the recorded daily caloric intake on the seventh day after initiation of PEG feeding or TPN, expressed as kcal/day. This variable was treated as a single day-7 daily value rather than cumulative or mean intake over the first 7 days. Nutritional route was classified as PEG or TPN, and no further source-level caloric decomposition was available. The exact clock-time window or timestamp rule used to derive the day-7 value was not available in the public dataset. Because individual body weight was not provided in the public analytic dataset, caloric intake could not be converted to kcal/kg/day. Day-7 caloric intake was analyzed primarily as a continuous variable per 100 kcal/day increase. For secondary categorical analysis and visualization, we used <900 versus ≥900 kcal/day as a rounded, exploratory threshold informed by the threshold analysis described below. The primary outcome was subsequent all-cause mortality among patients who survived beyond the day-7 landmark, with follow-up time calculated from the landmark point.

### 2.4 Covariates

Clinical and laboratory data were retrospectively extracted from medical records and comprised demographic information (age, sex), comorbid conditions (e.g., severe dementia, aspiration pneumonia) and baseline blood tests (serum albumin, hemoglobin and C-reactive protein). The route of artificial nutrition was chosen through discussion between the treating physician, the patient and/or their family, and the specific feeding regimen was prescribed at the clinician’s discretion. For analyses, laboratory results obtained within 7 days before initiation of PEG or TPN were used. Frailty was assessed using the Clinical Frailty Scale at the time of gastrostomy or at the start of parenteral nutrition. Severe frailty was defined as CFS ≥ 8 versus CFS < 8.

### 2.5 Statistical analysis

Categorical variables are presented as numbers and percentages, and continuous variables as means ± SD or medians with interquartile range, as appropriate. Baseline characteristics were compared using one-way ANOVA or the Kruskal–Wallis test for continuous variables, and the chi-square or Fisher’s exact test for categorical variables, as appropriate. Baseline characteristics were summarized according to day-7 caloric intake categories (<900 and ≥900 kcal/day) in the landmark cohort. We used Cox proportional hazards regression to estimate hazard ratios (HRs) and 95% confidence intervals (CIs) for the association between day-7 caloric intake and all-cause mortality in patients with dysphagia.

The proportional hazards assumption was evaluated before interpreting Cox regression models. Schoenfeld residuals were used to test whether the proportional hazards assumption was violated for the main exposure and covariates. To further evaluate potential confounding effects, we introduced covariates into a Cox regression model either in the basic model or sequentially removed them from the fully adjusted model one by one, comparing the resulting regression coefficients. Covariates leading to a change exceeding 10% in the original regression coefficients were preserved. Day-7 caloric intake was evaluated as the primary exposure in Cox proportional hazards models within the landmark cohort. It was analyzed both as a continuous variable, scaled per 100 kcal/day increase, and as a categorical variable using <900 kcal/day as the reference group. Four sequential models were constructed to examine the robustness of the association. Model 1 was unadjusted; Model 2 was adjusted for age and sex; Model 3 was further adjusted for nutritional route (PEG vs. TPN), severe dementia, aspiration pneumonia, serum albumin (Alb), and hemoglobin (Hb); Model 4 additionally included chronic heart failure (CHF), C-reactive protein (CRP), and Clinical Frailty Scale (CFS). These covariates were selected to partially account for demographic risk, nutritional route, comorbidity burden, nutritional status, systemic inflammation, anemia, and frailty. A multivariable-adjusted restricted cubic spline model with three knots was used to explore potential non-linear associations between day-7 caloric intake and subsequent mortality. When non-linearity was suggested, a two-piecewise Cox regression model was used to estimate the threshold point at which the association changed. Day-7 caloric intake was scaled per 100 kcal/day in Cox models, and the estimated threshold was converted back to kcal/day for reporting. A sensitivity analysis was additionally performed using the estimated threshold as an alternative categorical cutpoint. Because dichotomization may lead to information loss, the continuous Cox model and restricted cubic spline analysis were considered the primary evidence, whereas categorical analyses were used only for visualization, interpretability, and sensitivity assessment. Kaplan–Meier curves were generated to compare subsequent survival between patients with day-7 caloric intake <900 and ≥900 kcal/day, with differences assessed using the log-rank test. Subgroup and interaction analyses were conducted across age (<85 vs. ≥85 years), sex, nutritional route (PEG vs TPN), severe dementia, aspiration pneumonia, CHF and Alb (<3 vs. ≥3 g/dL), with forest plots summarizing results. During subgroup analysis, if the subgroup analysis variable is a categorical variable, it is excluded from the analysis. Effect modification was assessed using multivariable Cox regression and interactions tested via likelihood ratio tests.

To evaluate predictive performance, time-dependent ROC curves at 365 days were generated comparing a baseline Cox model (age, sex, nutritional route, severe dementia, aspiration pneumonia, serum albumin, hemoglobin) with an extended model including day-7 caloric intake, and the area under the curve (AUC) was calculated to quantify discrimination. Sensitivity analyses were performed using the fully adjusted Cox model. We repeated the analysis using a later outcome landmark at day 9 while retaining recorded day-7 caloric intake as the exposure; patients who did not survive beyond day 9 were excluded, and follow-up time was recalculated from day 9. We also excluded patients who died within 30 days after the day-7 landmark to assess whether the association was driven by imminent early mortality. All analyses were conducted utilizing R Statistical Software (Version 4.2.2, The R Foundation^1^) and Free Statistics analysis platform (Version 2.5.1, Beijing, China). A two-tailed *P* < 0.05 was considered statistically significant. A concise analytic workflow for reproducibility is provided in Supplementary Appendix 1.

## 3 Results

### 3.1 Study population, cohort derivation, and baseline characteristics

**FIGURE 1:**
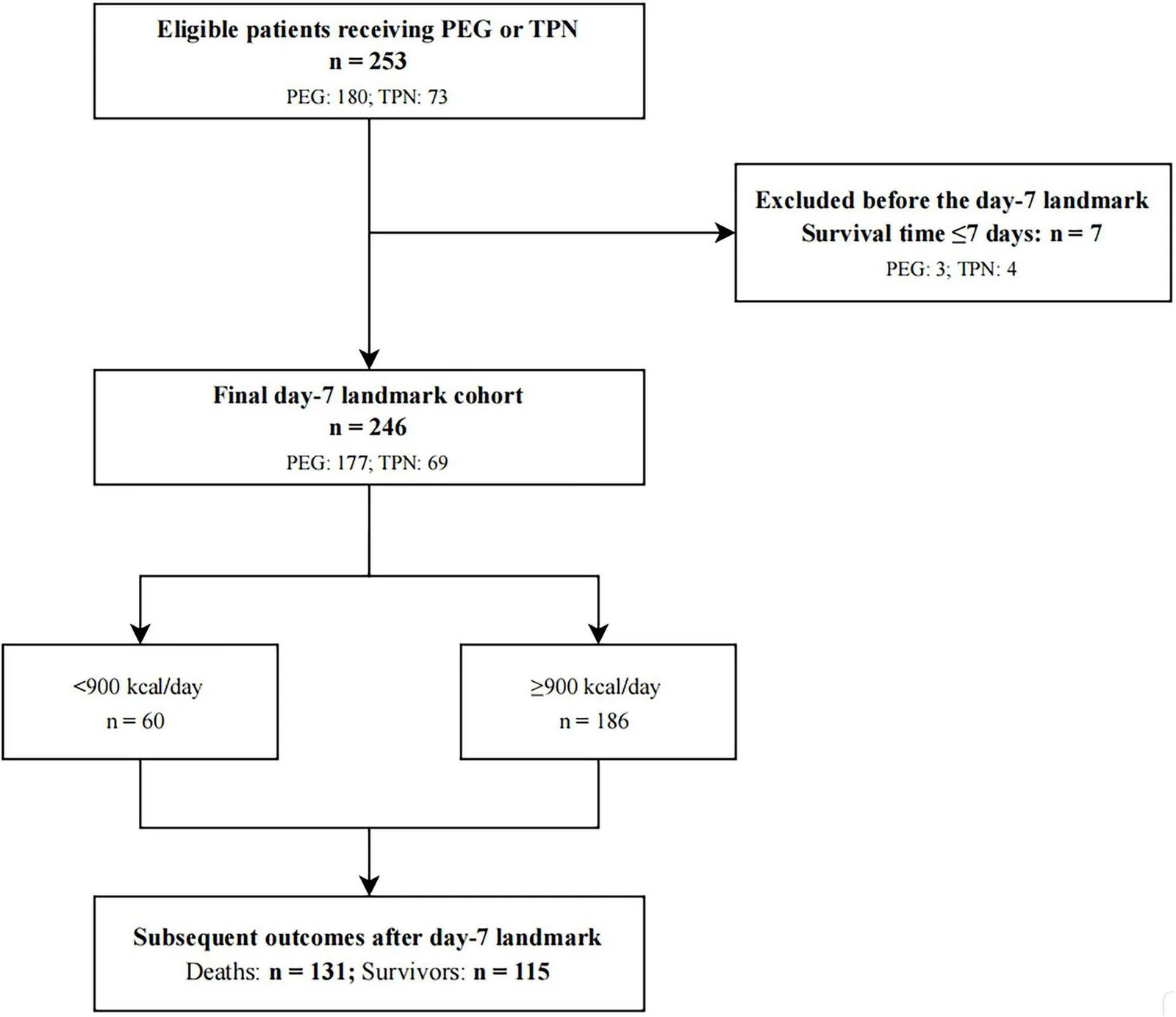
Study flow diagram for derivation of the day-7 landmark cohort. Patients with survival time ≤7 days were excluded because they did not survive beyond the day-7 landmark. The final landmark cohort included 246 patients. PEG, percutaneous endoscopic gastrostomy; TPN, total parenteral nutrition.

The derivation of the day-7 landmark cohort is shown in Figure 1. Of 253 eligible patients receiving PEG or TPN, 7 patients with survival time ≤ 7 days were excluded because they did not survive beyond the day-7 landmark, leaving 246 patients in the final landmark cohort. During subsequent follow-up, 131 deaths and 115 survivors were recorded. As summarized in Table 1, 60 patients had day-7 caloric intake < 900 kcal/day and 186 patients ≥900 kcal/day. The low-intake group was older (mean 87.3 vs. 81.5 years, *P* < 0.001) and showed a trend toward a higher proportion of females (71.7% vs. 57.5%, *P* = 0.051). Nutritional route differed markedly between caloric intake groups: PEG was used in 10 of 60 patients (16.7%) in the <900 kcal/day group and in 167 of 186 patients (89.8%) in the ≥900 kcal/day group. Severe dementia was more common in the low-intake group (66.7% vs. 31.2%, *P* < 0.001). No significant differences were observed in aspiration pneumonia or CHF. Nutritional and inflammatory indices differed significantly: albumin (2.8 vs. 3.2 g/dL, *P* < 0.001), hemoglobin (10.5 vs. 11.2 g/dL, *P* = 0.009) and CRP (median 2.0 vs. 0.7 mg/dL, *P* < 0.001). The low-intake group experienced shorter median survival (196.0 vs. 369.5 days, *P* < 0.001) and higher mortality (86.7% vs. 42.5%, *P* < 0.001).

**TABLE 1:** Characteristics of the day-7 landmark cohort according to day-7 caloric intake category.

Variables	Total	<900 kcal/day	≥900 kcal/day	*P*-value
*N*	246	60	186	
Age, years, mean ± SD	82.9 ± 9.4	87.3 ± 7.3	81.5 ± 9.5	<0.001
Sex, *n* (%)				0.051
Male	96 (39.0)	17 (28.3)	79 (42.5)	–
Female	150 (61.0)	43 (71.7)	107 (57.5)	–
Nutritional route, *n* (%)				<0.001
TPN	69 (28.0)	50 (83.3)	19 (10.2)	–
PEG	177 (72.0)	10 (16.7)	167 (89.8)	–
Severe dementia, *n* (%)				<0.001
No	148 (60.2)	20 (33.3)	128 (68.8)	–
Yes	98 (39.8)	40 (66.7)	58 (31.2)	–
Aspiration pneumonia, *n* (%)				0.127
No	156 (63.4)	43 (71.7)	113 (60.8)	–
Yes	90 (36.6)	17 (28.3)	73 (39.2)	–
CHF, *n* (%)				0.31
No	145 (58.9)	32 (53.3)	113 (60.8)	–
Yes	101 (41.1)	28 (46.7)	73 (39.2)	–
Alb, g/dL, mean ± SD	3.1 ± 0.6	2.8 ± 0.6	3.2 ± 0.6	<0.001
Hb, g/dL, mean ± SD	11.1 ± 2.0	10.5 ± 2.2	11.2 ± 1.9	0.009
CRP, mg/dL, median (IQR)	1.0 (0.3, 3.3)	2.0 (0.9, 4.3)	0.7 (0.2, 2.9)	<0.001
CFS, *n* (%)				0.209
<8	37 (15.0)	6 (10.0)	31 (16.7)	–
≥8	209 (85.0)	54 (90.0)	155 (83.3)	–
Survival, days, median (IQR)	315.0 (128.5, 618.8)	196.0 (52.8, 383.5)	369.5 (166.5, 668.5)	<0.001
Death, *n* (%)				<0.001
No	115 (46.7)	8 (13.3)	107 (57.5)	–
Yes	131 (53.3)	52 (86.7)	79 (42.5)	–

TPN, total parenteral nutrition; PEG, percutaneous endoscopic gastrostomy; CHF, chronic heart failure; Alb, albumin; Hb, hemoglobin; CRP, C-reactive protein; CFS, Clinical Frailty Scale; SD, standard deviation; IQR, interquartile range.

### 3.2 Association between day-7 caloric intake and subsequent mortality

The proportional hazards assumption was tested using Schoenfeld residuals, and no violation was observed (Supplementary Figure 1). Results from the multivariable Cox regression are presented in Table 2. Higher day-7 caloric intake was consistently associated with lower subsequent all-cause mortality across all models. When analyzed as a continuous variable, each 100 kcal/day increase in day-7 caloric intake was associated with a lower risk of subsequent all-cause mortality in the crude model (HR 0.78, 95% CI 0.70–0.86; *P* < 0.001) and remained significant in the fully adjusted model (HR 0.82, 95% CI 0.72–0.93; *P* = 0.002). Similar findings were observed in the categorical analysis. Compared with patients receiving <900 kcal/day, those with ≥900 kcal/day had significantly lower mortality risk in the crude model (HR 0.31, 95% CI 0.22–0.45; *P* < 0.001) and in the fully adjusted model (HR 0.47, 95% CI 0.26–0.83; *P* = 0.010), suggesting that the association remained after multivariable adjustment.

**TABLE 2:** Association between day-7 caloric intake and subsequent all-cause mortality in the landmark cohort.

Variables	Model 1		Model 2		Model 3		Model 4	
	HR (95% CI)	*P*	HR (95% CI)	*P*	HR (95% CI)	*P*	HR (95% CI)	*P*
Day-7 caloric intake (per 100 kcal/day)	0.78 (0.70–0.86)	<0.001	0.76 (0.68–0.85)	<0.001	0.83 (0.74–0.94)	0.003	0.82 (0.72–0.93)	0.002
Day-7 caloric intake category								
<900 kcal/day	1 (ref)	–	1 (ref)	–	1 (ref)	–	1 (ref)	–
≥900 kcal/day	0.31 (0.22–0.45)	<0.001	0.29 (0.20–0.43)	<0.001	0.47 (0.26–0.84)	0.011	0.47 (0.26–0.83)	0.010

Model 1 was unadjusted. Model 2 was adjusted for age and sex. Model 3 was adjusted for age, sex, nutritional route (PEG vs. TPN), severe dementia, aspiration pneumonia, Alb, and Hb. Model 4 was further adjusted for CHF, CRP, and CFS. HR, hazard ratio; CI, confidence interval; PEG, percutaneous endoscopic gastrostomy; TPN, total parenteral nutrition; Alb, albumin; Hb, hemoglobin; CHF, chronic heart failure; CRP, C-reactive protein; CFS, Clinical Frailty Scale.

Following multivariable adjustment in Model 4, restricted cubic spline analysis suggested a non-linear inverse association between day-7 caloric intake and subsequent mortality (*P* for overall association = 0.002; *P* for non-linearity = 0.031) (Supplementary Figure 2). Two-piecewise Cox regression estimated an inflection point of approximately 870 kcal/day, below which the inverse association was stronger (Supplementary Table 1A). Using 870 kcal/day as an alternative categorical cutpoint yielded consistent results in the fully adjusted model (HR, 0.48; 95% CI, 0.27–0.84; *P* = 0.011; Supplementary Table 1B). Additional sensitivity analyses using a day-9 outcome landmark and excluding deaths within 30 days after the day-7 landmark also yielded consistent results (Supplementary Table 2). The fully adjusted HR was 0.83 (95% CI, 0.73–0.94; *P* = 0.004) in the day-9 landmark analysis and 0.80 (95% CI, 0.69–0.93; *P* = 0.003) after excluding early post-landmark deaths.

### 3.3 Kaplan–Meier survival analysis

**FIGURE 2:**
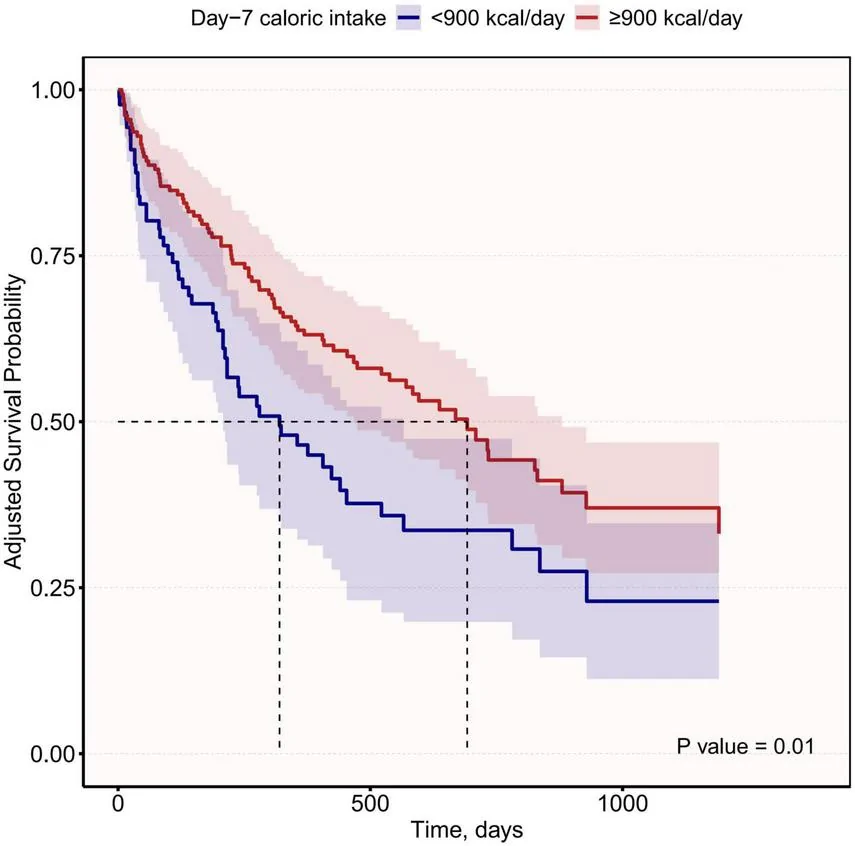
Multivariable-adjusted survival curves according to day-7 caloric intake category (<900 vs. ≥900 kcal/day). Curves were adjusted for age, sex, nutritional route, severe dementia, aspiration pneumonia, CHF, Alb, Hb, CRP, and CFS. PEG, percutaneous endoscopic gastrostomy; CHF, chronic heart failure; Alb, albumin; Hb, hemoglobin; CRP, C-reactive protein; CFS, Clinical Frailty Scale.

Multivariable-adjusted Kaplan–Meier survival curves demonstrated higher subsequent survival in patients with day-7 caloric intake ≥900 kcal/day compared with those with <900 kcal/day (*P* = 0.01) (Figure 2). This result was consistent with the multivariable Cox regression analysis, supporting an inverse association between higher day-7 caloric intake and subsequent all-cause mortality in the landmark cohort.

### 3.4 Subgroup analyses and predictive performance

**FIGURE 3:**
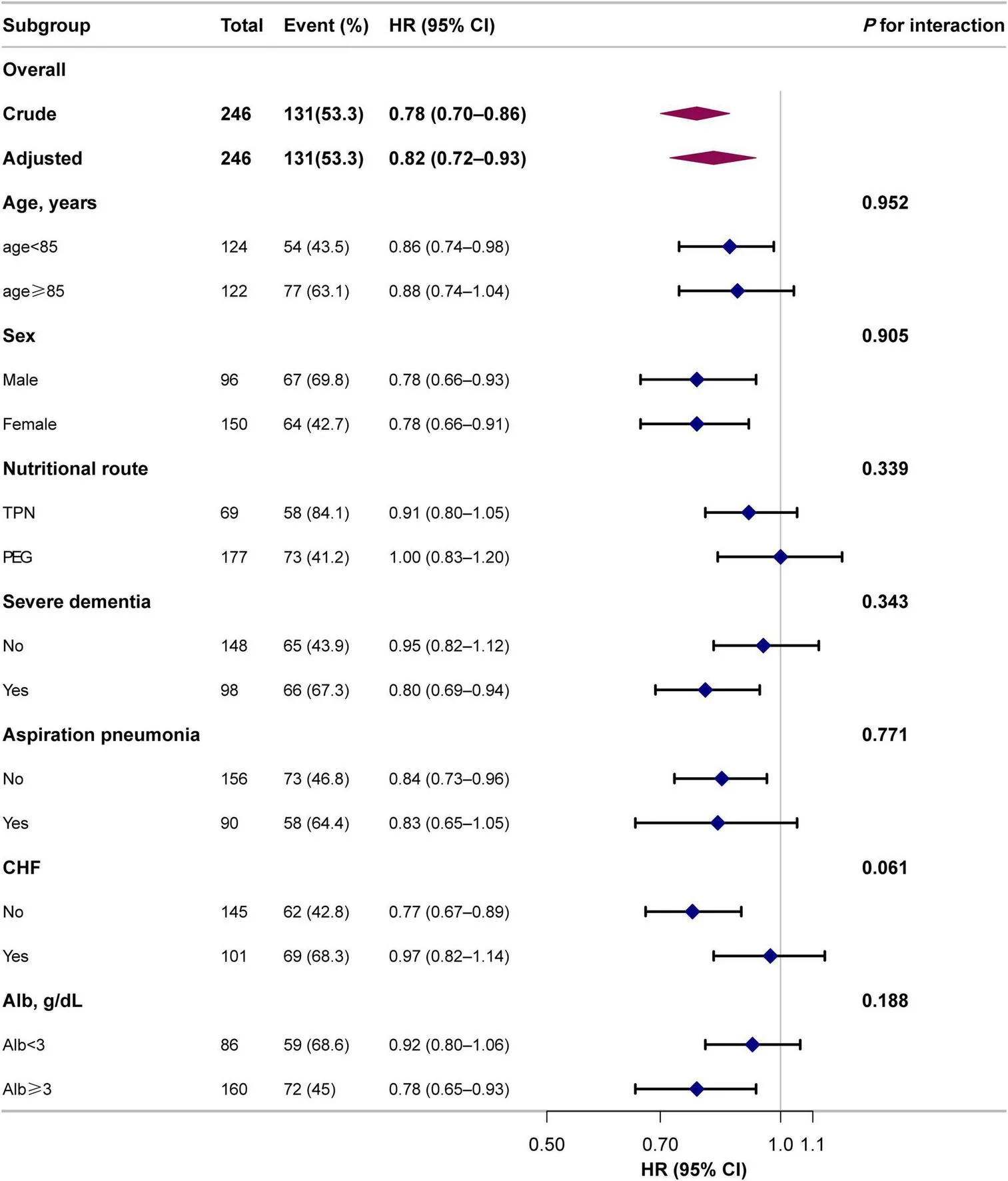
Subgroup analysis of the association between day-7 caloric intake and subsequent all-cause mortality in the landmark cohort. HR, hazard ratio; CI, confidence interval; PEG, percutaneous endoscopic gastrostomy; TPN, total parenteral nutrition; CHF, chronic heart failure; Alb, albumin.

**FIGURE 4:**
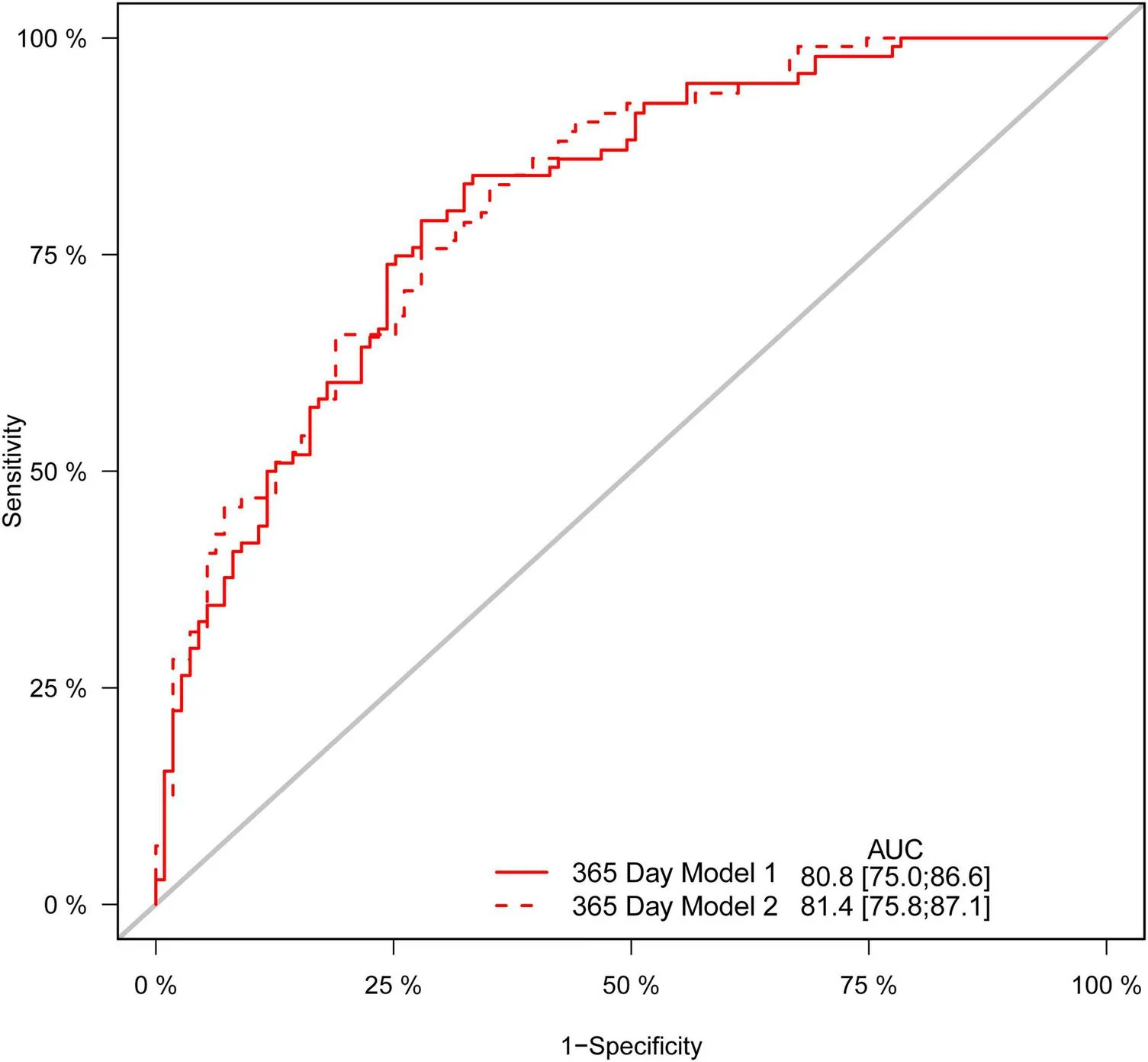
Time-dependent receiver operating characteristic (ROC) curves at 365 days after the landmark time. The baseline model is compared with the extended model including day-7 caloric intake.

Subgroup analyses showed that the inverse association between day-7 caloric intake and subsequent all-cause mortality was generally consistent across clinically relevant strata, including age (<85 vs. ≥ 85 years), sex, nutritional route (PEG vs. TPN), severe dementia, aspiration pneumonia, chronic heart failure, and baseline Alb (<3 vs. ≥3 g/dL). No statistically significant interactions were observed (all *P* for interaction > 0.05; Figure 3). In particular, the interaction between day-7 caloric intake and nutritional route was not statistically significant (*P* for interaction = 0.339), suggesting no clear evidence that the association differed between PEG and TPN patients. For 1-year mortality prediction, the AUC of the extended model including day-7 caloric intake was numerically higher than that of the baseline model (0.814 vs. 0.808), suggesting modest improvement in discrimination for subsequent mortality in the landmark cohort (Figure 4).

## 4 Discussion

In this landmark cohort of older Japanese patients with dysphagia receiving artificial nutritional support, higher day-7 caloric intake was consistently associated with lower subsequent all-cause mortality. This association was robust when caloric intake was analyzed both continuously and categorically. Restricted cubic spline analysis suggested a non-linear inverse relationship, and Kaplan–Meier curves demonstrated better survival among patients receiving ≥900 kcal/day. Subgroup analyses confirmed the consistency of this association across clinically relevant strata, with no significant interactions observed. Moreover, the extended model including day-7 caloric intake showed a slightly higher time-dependent AUC compared with the baseline clinical model, indicating that this dynamic nutritional marker may provide modest additional discrimination beyond conventional baseline factors.

This study extends previous research by evaluating an early post-procedural dynamic marker rather than solely relying on static baseline indicators. Prior secondary analyses of the same Japanese dysphagia cohort demonstrated that serum albumin, the C-reactive protein-to-albumin ratio, prognostic nutritional index, controlling nutritional status score, and related inflammatory-nutritional indices were predictive of mortality (14, 15, 19–21), highlighting the central role of malnutrition, inflammation, and immune dysfunction in older patients with dysphagia. However, baseline biomarkers cannot fully capture early responses to artificial nutritional support. In contrast, day-7 caloric intake reflects practical achievement of nutritional support and integrates information on feeding tolerance, infection burden, clinical stability, catabolic stress, functional reserve, and care goals. The landmark design strengthens the methodological rigor of this analysis: only patients surviving to day 7 were included, follow-up was recalculated from the landmark time, and early deaths were excluded, thereby ensuring that exposure assessment preceded outcome measurement and avoiding immortal time bias. Consequently, among patients who survived to the early reassessment point, lower day-7 caloric intake identified those at higher risk of subsequent mortality. Landmark analysis is widely applied to address this type of time-dependent exposure in survival studies (17, 18, 22).

Several biological mechanisms may underlie the observed inverse association between day-7 caloric intake and subsequent mortality in older patients with dysphagia. Low early energy intake likely reflects unresolved clinical instability, impaired feeding tolerance, ongoing infection, or diminished gastrointestinal and metabolic function, each of which can contribute to inadequate energy availability during critical recovery phases (23, 24). Inadequate energy provision exacerbates protein-energy malnutrition and sarcopenia, leading to accelerated loss of skeletal muscle mass and strength, impaired immune competence, delayed wound healing, and heightened susceptibility to infections and aspiration-related complications—all established predictors of adverse outcomes in geriatric populations (25–27). Higher recorded caloric intake by day 7 may indicate more successful stabilization of nutritional support, better feeding tolerance, and greater physiological reserve, rather than directly proving that increasing caloric delivery itself reduces mortality. Evidence from observational studies indicates that higher total energy intake is associated with reduced frailty and mortality risk in older adults, independent of specific nutrient subcomponents, underscoring the prognostic relevance of overall energy status in aging and disease contexts (28). Clinical nutrition guidelines recommend individualized energy targets in older patients based on nutritional status, disease severity, activity level, and tolerance, with values such as 27–30 kcal/kg/day often used as a reference rather than fixed prescription (29). Thus, in the present landmark cohort, day-7 caloric intake should be interpreted not as a prescriptive dose but as an integrated marker of early nutritional achievement, feeding competence, and treatment tolerance that captures dynamic physiological responses to artificial nutritional support.

The non-linear inverse association observed in the restricted cubic spline analysis is clinically plausible. Mortality risk appeared to decrease more steeply at lower caloric intake levels, likely reflecting unresolved disease severity, impaired feeding tolerance, or persistent infection, and then plateaued at higher intake levels, where further increases provided limited incremental prognostic information. The two-piecewise Cox regression model estimated a threshold of approximately 870 kcal/day. The 900 kcal/day cutoff used in the categorical analysis should be interpreted only as a rounded, clinically interpretable value close to the estimated threshold, rather than as a definitive biological cutoff or nutritional target. This interpretation is consistent with ESPEN guidelines recommending individualized energy intake in older adults rather than a fixed caloric target (16). Subgroup analyses further reinforced the robustness of these findings, with the inverse association between day-7 caloric intake and mortality generally consistent across age, sex, nutritional route, severe dementia, aspiration pneumonia, and baseline albumin strata, with no significant interactions observed. This consistency is clinically relevant, as patients received either PEG or TPN, and aligns with findings from the original Japanese cohort, in which PEG was associated with longer survival, higher risk of severe pneumonia, and lower risk of sepsis compared with TPN (7). Collectively, these results suggest that, regardless of the initial nutrition route, achieved caloric intake by day 7 provides additional prognostic information, supporting the use of day 7 as a practical reassessment point after initiation of artificial nutrition.

The prediction analysis also has clinical implications. The baseline model included age, sex, nutritional route, severe dementia, aspiration pneumonia, serum albumin, and hemoglobin. The observed increase in time-dependent AUC after adding day-7 caloric intake suggests that this early dynamic marker may modestly improve discrimination beyond baseline clinical status, indicating that early nutritional achievement provides additional prognostic information for subsequent mortality. These findings highlight that risk assessment in older dysphagic patients should not be limited to the time of PEG or TPN initiation; the first week of artificial nutrition represents a clinically meaningful window for reassessing prognosis, optimizing nutritional plans, and communicating realistic expectations with patients and families. Time-dependent ROC analysis provides a robust approach to quantify model discrimination for survival outcomes and is increasingly recommended in geriatric prognostic studies (30).

This study has several limitations. First, it is a secondary analysis of a single-center retrospective cohort, which may constrain generalizability. Second, most participants had severe dysphagia requiring PEG or TPN, and the findings may not apply to patients with mild or transient post-stroke dysphagia, community-dwelling older adults, or those receiving oral nutritional supplementation alone. Third, although the landmark design enhanced temporal validity, the analysis was conditional on survival beyond day 7; therefore, the findings apply only to patients who survived to the landmark. Residual confounding cannot be excluded, as low day-7 caloric intake may reflect unmeasured illness severity, infection, feeding intolerance, treatment interruption, or end-of-life care preferences. Sensitivity analyses using a later outcome landmark and excluding early post-landmark deaths yielded consistent results, but they cannot fully eliminate survivor selection or residual confounding. Fourth, day-7 caloric intake was available only as a single recorded kcal/day value. The public dataset did not provide more granular timing information; therefore, we could not determine whether this value corresponded to a calendar day, a clinical 24-h recording period, or another institution-specific accounting interval. Individual body weight was unavailable, precluding conversion of caloric intake to kcal/kg/day. Component-level information on oral, enteral, parenteral, intravenous dextrose, medication-derived, supplemental, or other hidden caloric sources was also unavailable; therefore, we could not determine which sources were included, evaluate their individual contributions, or reconstruct the calorie-summing formula and conversion constants used in the original dataset. The dataset also did not indicate whether this value reflected prescribed calories or actually delivered intake, nor whether missed feeds, feeding holds, vomiting or regurgitation, tube dislodgement, partial feeds, or other interruptions were excluded, adjusted for, or left as recorded. Route-specific measurement error may have occurred because TPN calories may be more directly derived from infusion prescriptions or records, whereas PEG feeding may be more susceptible to interruption, intolerance, reflux, tube-related problems, or incomplete delivery. Such error could have attenuated the observed association or obscured route-specific differences, but its direction and magnitude could not be quantified. In addition, repeated day-7 measurements of inflammatory markers such as CRP, body weight or weight change, swallowing function, and the dynamic status of the underlying disease causing dysphagia were not available in the public dataset; therefore, we could not compare the prognostic value of day-7 caloric intake with other day-7 clinical or laboratory reassessment markers. The absence of longitudinal nutritional data also precluded evaluation of cumulative intake, protein provision, and temporal nutritional trajectories. Fifth, the timing and standardization of laboratory and clinical assessments could not be fully controlled. Finally, while internal model comparisons indicated a slight improvement in discrimination after incorporating day-7 caloric intake, external validation in independent cohorts is necessary before this measure can be adopted as a formal prognostic marker.

## 5 Conclusion

Higher day-7 caloric intake was associated with lower subsequent all-cause mortality in older Japanese patients with dysphagia who survived beyond the day-7 landmark of artificial nutritional support. Incorporating day-7 caloric intake modestly improved prediction beyond baseline clinical factors, supporting its potential as an early dynamic prognostic marker. Future multicenter prospective studies are warranted to validate these findings and evaluate whether early individualized nutritional interventions can improve survival and patient-centered outcomes.

## Data Availability

Publicly available datasets were analyzed in this study. This data can be found here: https://datadryad.org/search?q=10.5061%2Fdryad.gg407h1.

## Footnotes

1
http://www.R-project.org
