# Supplementary material for: Day-7 caloric intake as an early dynamic prognostic marker for subsequent mortality in older patients with dysphagia: a landmark cohort analysis
Source: Front Nutr. 2026 Aug 26;13:1887101. doi: 10.3389/fnut.2026.1887101 (PMC13553383; doi:10.3389/fnut.2026.1887101)
Supplement: Supplementary file 1 [file Table_1.docx]

# Supplementary Appendix 1. Analytic workflow for reproducibility

This study was a secondary analysis of a publicly available patient-level analytic dataset. Because the released dataset rather than the original hospital database was used, SQL extraction criteria were not available.

The analytic workflow was as follows:

1. **Construct the day-7 landmark cohort**
   Eligible patients: n = 253.
   Patients with survival time ≤7 days were excluded because they did not survive beyond the day-7 landmark.
   Final landmark cohort: n = 246.
   Follow-up time was recalculated as:
   **follow-up time = original survival time − 7**.
2. **Define exposure and outcome**
   The primary exposure was recorded day-7 caloric intake, expressed as kcal/day.
   It was analyzed as a continuous variable per 100 kcal/day increase and secondarily as <900 versus ≥900 kcal/day.
   The outcome was subsequent all-cause mortality after the day-7 landmark.
3. **Specify Cox regression models**
   Cox proportional hazards models were fitted sequentially.
   Model 1 was unadjusted.
   Model 2 adjusted for age and sex.
   Model 3 further adjusted for nutritional route (PEG vs TPN), severe dementia, aspiration pneumonia, albumin, and hemoglobin.
   Model 4 additionally adjusted for chronic heart failure, C-reactive protein, and Clinical Frailty Scale.
4. **Additional and sensitivity analyses**
   Restricted cubic spline and two-piecewise Cox regression were used to evaluate the dose-response pattern and threshold.
   Subgroup and interaction analyses were performed across clinically relevant strata, including nutritional route.
   Sensitivity analyses included a later outcome landmark at day 9 while retaining day-7 caloric intake as the exposure, and exclusion of deaths occurring within 30 days after the day-7 landmark.
5. **Missing data**
   In the final landmark cohort, there were no missing values for the exposure, outcome, survival time, or covariates included in the fully adjusted model; therefore, multiple imputation was not required.
