# Supplementary material for: Day-7 caloric intake as an early dynamic prognostic marker for subsequent mortality in older patients with dysphagia: a landmark cohort analysis
Source: Front Nutr. 2026 Aug 26;13:1887101. doi: 10.3389/fnut.2026.1887101 (PMC13553383; doi:10.3389/fnut.2026.1887101)
Supplement: Supplementary file 2 [file Table_2.docx]

Supplementary Material

**Supplementary Figure S1.** Assessment of the proportional hazards assumption for day-7 caloric intake using scaled Schoenfeld residuals. The upper panel presents day-7 caloric intake as a continuous variable (kcal/day), and the lower panel presents day-7 caloric intake as a categorical variable. The x-axis indicates follow-up time in days, and the y-axis indicates the time-varying regression coefficient β(t). Open circles represent scaled Schoenfeld residuals, the solid red line represents the smoothed estimate of β(t), and the dashed red lines represent the approximate 95% confidence bands.

**Supplementary Figure S2. Restricted cubic spline curve for the association between day-7 caloric intake and hazard ratio.** The red solid line represents the estimated hazard ratio, and the shaded area represents the 95% confidence interval.
