# Supplementary material for: Day-7 caloric intake as an early dynamic prognostic marker for subsequent mortality in older patients with dysphagia: a landmark cohort analysis
Source: Front Nutr. 2026 Aug 26;13:1887101. doi: 10.3389/fnut.2026.1887101 (PMC13553383; doi:10.3389/fnut.2026.1887101)
Supplement: Supplementary file 3 [file Table_3.docx]

**Supplementary Table 1A.** Threshold analysis using two-piecewise Cox regression.

| **Day-7 caloric intake** | **Adjusted HR (95% CI)** | ***P* value** |
| --- | --- | --- |
| Estimated inflection point | 8.70 per 100 kcal/day scale, approximately 870 kcal/day |  |
| <870 kcal/day | 0.66 (0.51–0.86) | 0.002 |
| ≥870 kcal/day | 0.99 (0.83–1.20) | 0.946 |
| Likelihood Ratio test |  | 0.013 |

HR, hazard ratio; CI, confidence interval. Day-7 caloric intake was scaled per 100 kcal/day before modeling; therefore, the estimated inflection point of 8.70 corresponds to approximately 870 kcal/day. HRs represent the change in subsequent mortality risk per 100 kcal/day increase within each segment. The model was adjusted for age, sex, nutritional route, severe dementia, aspiration pneumonia, chronic heart failure, albumin, hemoglobin, C-reactive protein, and Clinical Frailty Scale.

**Supplementary Table 1B.** Sensitivity analysis using 870 kcal/day as an alternative categorical cutpoint.

| **Variables** | **Model 1** | | **Model 2** | | **Model 3** | | **Model 4** | |
| --- | --- | --- | --- | --- | --- | --- | --- | --- |
|  | **HR (95% CI)** | ***P*** | **HR (95% CI)** | ***P*** | **HR (95% CI)** | ***P*** | **HR (95% CI)** | ***P*** |
| Day-7 caloric intake category | |  |  |  |  |  |  |  |
| <870 kcal/day | 1(Ref) |  | 1(Ref) |  | 1(Ref) |  | 1(Ref) |  |
| ≥870 kcal/day | 0.34 (0.23-0.48) | <0.001 | 0.31 (0.21-0.45) | <0.001 | 0.50 (0.28-0.89) | 0.019 | 0.48 (0.27-0.84) | 0.011 |

Model 1 was unadjusted. Model 2 was adjusted for age and sex. Model 3 was adjusted for age, sex, nutritional route (PEG vs TPN), severe dementia, aspiration pneumonia, Alb, and Hb. Model 4 was further adjusted for CHF, CRP, and CFS. The <870 versus ≥870 kcal/day cutpoint was used as a sensitivity analysis based on the estimated threshold from the two-piecewise Cox regression model. HR, hazard ratio; CI, confidence interval; PEG, percutaneous endoscopic gastrostomy; TPN, total parenteral nutrition; Alb, albumin; Hb, hemoglobin; CHF, chronic heart failure; CRP, C-reactive protein; CFS, Clinical Frailty Scale.

**Supplementary Table 2.** Sensitivity analyses using a later outcome landmark and excluding early post-landmark deaths.

| **Sensitivity analysis** | **N** | **Events(%)** | **Exposure** | **Adjusted HR** | **95% CI** | ***P-value*** |
| --- | --- | --- | --- | --- | --- | --- |
| Day-9 outcome landmark | 245 | 130 (53.1) | Per 100 kcal/day increase | 0.83 | 0.73-0.94 | 0.004 |
| Excluding deaths within 30 days after day-7 landmark | 228 | 113 (49.6) | Per 100 kcal/day increase | 0.80 | 0.69-0.93 | 0.003 |

Day-7 caloric intake was retained as the exposure in all sensitivity analyses. Models were fully adjusted for age, sex, nutritional route, severe dementia, aspiration pneumonia, chronic heart failure, albumin, hemoglobin, C-reactive protein, and Clinical Frailty Scale.
